# Leg pain location and neurological signs relate to outcomes in primary care patients with low back pain

**DOI:** 10.1186/s12891-017-1495-3

**Published:** 2017-03-31

**Authors:** Lisbeth Hartvigsen, Lise Hestbaek, Charlotte Lebouef-Yde, Werner Vach, Alice Kongsted

**Affiliations:** 1 Department of Sports Science and Clinical Biomechanics, University of Southern Denmark, Odense, Denmark; 2 Nordic Institute of Chiropractic and Clinical Biomechanics, Odense, Denmark; 3 Research Department, Spine Center of Southern Denmark, Hospital Lillebælt, Middelfart, Denmark; 4 Institute for Regional Health Research, University of Southern Denmark, Odense, Denmark; 5 Institute for Medical Biometry and Statistics, Faculty of Medicine and Medical Center, University of Freiburg, Freiburg, Germany

**Keywords:** Classification, Quebec Task Force classification, Cohort studies, Low back pain, Primary care, Radiculopathy, Referred leg pain, Sciatica

## Abstract

**Background:**

Low back pain (LBP) patients with related leg pain and signs of nerve root involvement are considered to have a worse prognosis than patients with LBP alone. However, it is unclear whether leg pain location above or below the knee and the presence of neurological signs are important in primary care patients. The objectives of this study were to explore whether the four Quebec Task Force categories (QTFC) based on the location of pain and on neurological signs have different characteristics at the time of care seeking, whether these QTFC are associated with outcome, and if so whether there is an obvious ranking of the four QTFC on the severity of outcomes.

**Method:**

Adult patients seeking care for LBP in chiropractic or general practice were classified into the four QTFC based on self-reported information and clinical findings. Analyses were performed to test the associations between the QTFC and baseline characteristics as well as the outcomes global perceived effect and activity limitation after 2 weeks, 3 months, and 1 year and also 1-year trajectories of LBP intensity.

**Results:**

The study comprised 1271 patients; 947 from chiropractic practice and 324 from general practice. The QTFC at presentation were statistically significantly associated with most of the baseline characteristics, with activity limitation at all follow-up time points, with global perceived effect at 2 weeks but not 3 months and 1 year, and with trajectories of LBP. Severity of outcomes in the QTFC increased from LBP alone, across LBP with leg pain above the knee and below the knee to LBP with nerve root involvement. However, the variation within the categories was considerable.

**Conclusion:**

The QTFC identify different LBP subgroups at baseline and there is a consistent ranking of the four categories with respect to outcomes. The differences between outcomes appear to be large enough for the QTFC to be useful for clinicians in the communication with patients. However, due to variation of outcomes within each category individuals’ outcome cannot be precisely predicted from the QTFC alone. It warrants further investigation to find out if the QTFC can improve existing prediction tools and guide treatment decisions.

**Electronic supplementary material:**

The online version of this article (doi:10.1186/s12891-017-1495-3) contains supplementary material, which is available to authorized users.

## Background

Low back pain (LBP) is the leading cause of disability worldwide contributing more than 10 of total years lived with disability [1] and the cost for society is huge. In the United States alone the estimated direct medical cost for all back-related conditions was $253 billion in 2009 to 2011 and back pain resulted in more than 290 million lost workdays [2]. Most of the money is spent on the small minority of patients with persistent work disability [3], and the need for prognostic assessment is highlighted in evidence-based guidelines for nonspecific low back pain in primary care [4]. Feasible assessment tools to identify prognostic indicators are needed that can facilitate clinical decision-making with the ultimate goal of preventing persistent problems and reducing costs.

Many attempts have been made to develop such back pain classification systems and screening tools [5–7]. One tool is the Quebec Task Force (QTF) classification of spinal disorders, proposed in 1987 [7]. The classification in its original form includes 11 categories. Categories 1 to 3 are based on the location of pain (‘LBP alone’, ‘LPB + leg pain above the knee’, ‘LBP + leg pain below the knee’), whereas category 4 requires the presence of signs of nerve root involvement (NRI) in the clinical examination (‘LBP + NRI’). QTF categories 5 to 7 are based on the results of imaging, categories 8 to 10 on the response to treatment, and category 11 is based on paraclinical tests.

Although the QTF classification was originally developed as a guideline to the management of patients with spinal disorders, the QTF categories 1 to 4, which are based on pain distribution and signs of NRI, have been evaluated in different settings for their discriminative and predictive ability [8–13]. These categories have been shown to differ on baseline patient profiles, generally with increasing severity from category 1 to 4 [11, 12] and also on outcomes [10, 11, 13, 14]. In workers on sick leave, leg pain below the knee and signs of NRI were shown to be strong predictors of prolonged disability and greater back-related costs [9]. Signs of NRI has been associated with greater improvement, but at the same time poorer absolute outcomes than LBP +/− leg pain above or below the knee in patients in a secondary care setting [13]. Two primary care studies found that radiating leg pain was associated with more severe pain and disability than ‘LBP alone’ both at presentation and after 6 months, and it was worse for patients with pain radiating below the knee compared to patients with pain above the knee [14, 15]. None of these studies investigated neurological signs as prognostic factors.

A systematic review of the literature on LBP-related leg pain reported consistent evidence for worse health outcomes and increased utilization of health care with radiation of leg pain below the knee and with neurological findings [16]. However, in a systematic review concerning non-surgically treated sciatica, findings on neurological deficit as a significant prognostic factor of poor outcome were inconsistent [17].

Although several studies have shown LBP-related leg pain to be a poor prognostic factor, studies often either fail to define leg pain/sciatica/radiculopathy or use LBP with leg pain below the knee as a proxy for nerve root pain [14]. No primary care studies have investigated whether LBP with leg pain below the knee has as different prognosis than LBP with nerve root involvement. Clinical guidelines for LBP recommend that, as part of the initial clinical history, pain distribution should be addressed and that the initial examination should include a neurological screening [4, 18]. However, the impact of both pain distribution in itself and neurological findings as well as the clinical relevance of differentiating between each of the four QTF categories remain to be investigated in primary care.

The objectives of this study are to explore 1) if the four QTF categories (‘LBP alone’, ‘LBP + leg pain above the knee’, ‘LBP + leg pain below the knee’, and ‘LBP + NRI’) have different characteristics at the time of care seeking; 2) whether the QTF categories are associated with the outcomes global perceived effect (GPE) and activity limitation after 2 weeks, 3 months, and 1 year and also 1-year trajectories of LBP intensity; if so, 3) whether this association is independent of socio-demographic factors; and lastly, 4) if there is an obvious ranking of the four QTF categories on the severity of outcomes. In studying the degree of association, we focus on the differences in expected outcomes i.e. the separative capacity of the QTF categories [19] and whether they reach a clinically relevant degree. In our opinion this requires that the group differences are large enough to add meaningful and useful information to patients or clinicians about the average future course of patients in the different QTF categories.

## Methods

### Design and setting

Patients with LBP were recruited by chiropractors and general practitioners to participate in a prospective observational study.

Thirty-six chiropractors (17 clinics), in a research network of the Nordic Institute of Chiropractic and Clinical Biomechanics that were geographically spread across Denmark, agreed to include consecutive patients with LBP from September 2010 till January 2012. Participating chiropractors attended a 1-day course introducing study procedures and a research assistant visited all clinics prior to study start to ensure that the clinical examination procedures related to the study were adequately standardized.

All 800 general practitioners in the Region of Southern Denmark were invited to participate in a quality development initiative by the Audit Project Odense [19]. Of the general practitioners, 88 agreed to participate and include patients over 10 weeks in 2011. No attempt was made to standardize the clinical examination procedures in general practice. Patient recruitment has been described in more detail elsewhere [19, 20].

As the study was observational, treatment was not affected by participation. Patients received ‘usual care’ from their chiropractor/general practitioner.

### Participants

Patients were invited to participate if they sought care for LBP with or without leg pain, were 18 to 65 years of age, could read and understand Danish, had access to a mobile phone and were able to use text messaging (as one of the outcome measures was based on responses to text messages). Patients were not included if pathology or inflammatory pain was suspected or if their condition required acute referral for surgery. Chiropractic patients were also excluded if they were pregnant or if they had had more than one health care consultation for their LBP within the previous 3 months.

### Data collection

Chiropractic patients completed a baseline questionnaire in the reception area before the first consultation and returned it in a sealed envelope to the clinic secretary who sent it to the research unit. Patients consulting a general practitioner were given an envelope with information on the project and a baseline questionnaire following the first consultation. If they consented to participate, they were asked to complete the questionnaire at home and send it to the research unit in a prepaid envelope. In both settings, the included patients were given the 2-week follow-up questionnaire and a prepaid envelope at the initial consultation.

The clinical examination by the chiropractor followed a standardized examination protocol thoroughly described elsewhere [21] including questions on pain localization (‘LPB alone’, ‘LBP + leg pain above the knee’, ‘LBP + leg pain below the knee’) and a lumbar neurological examination (straight leg raise, femoral nerve stretch test, muscle strength, deep tendon reflexes, and sensitivity to touch or pinprick). On the basis of these variables, the chiropractors classified patients according to the QTF categories 1 to 4.

Data collection in general practice consisted of a practitioner-completed questionnaire including questions on pain localization (‘LPB alone’, ‘LPB + leg pain above the knee’, ‘LBP + leg pain below the knee’) and a yes/no question on the presence of abnormal neurological findings. On the basis of these variables, we classified patients according to the QTF categories 1 to 4.

Follow-up questionnaires were posted to the participants 3 months and 1 year after the initial consultation and non-responders were contacted by phone. Each Sunday for 52 weeks, patients received an SMS question asking about LBP intensity during the preceding week. Patients replied to the SMS question by sending a return text message that went directly into a data file accessible to the researchers.

### Baseline characteristics

#### The Quebec Task Force classification

The QTF categories 1–4 (‘LPB alone’, ‘LBP + leg pain above the knee’, ‘LBP + leg pain below the knee’, or ‘LBP + NRI’) [7].

To investigate whether the four QTF categories had different characteristics at the time of care seeking the following variables were considered:

#### Socio-demographics

Information on age, sex, physical work load (mainly sitting, sitting and walking, light physical work, or hard physical work), sick leave (the proportion reporting any days off work due to LBP within the previous month), educational level (no qualification, vocational training, higher education of < 3 years, higher education of 3–4 years, or higher education of >4 years), and activity limitation (Roland Morris Disability Questionnaire (RMDQ) proportional score (0–100) [22]).

#### LBP characteristics

Duration of pain (<2 weeks, 2–4weeks, 1–3months, or >3 months), previous LBP episodes (0, 1–3, or > 3), LBP last year (≤30 days or > 30 days), LBP intensity (typical intensity of back pain during the last week measured on a Numeric Rating Scale (NRS) 0–10 (0: no pain, 10: worst imaginable pain) [23]), leg pain intensity (typical intensity of leg pain during the last week measured on NRS 0–10 (0: no pain, 10: worst imaginable pain) [23]).

#### Psychological factors

Recovery expectations 0–10 (“How likely do you think it is that you will be fully recovered in 3 months?” 0: no chance, 10: high chance) [24]), depressive symptoms (Major Depression Inventory 0–50, sum score) [25], *Fear-Avoidance Beliefs Questionnaire* (*FABQ*) physical activity scale (0–24, sum score), *FABQ* work scale (0–42, sum score) [26].

#### General health

Self-perceived general health measured by the EuroQol-5D Visual Analogue Scale 0–100 (0: worst imaginable health state, 100: best imaginable health state) [27].

#### STarT Back Tool

The STarT Back Tool (SBT) (three prognostic profiles: low, medium, and high-risk groups for persisting LBP disability) [28].

### Outcome measures

To investigate whether the QTF categories were associated with outcome and, if so, whether there was a obvious ranking of the four QTF categories on the severity of outcomes the following outcome measures were used:

#### Global perceived effect (GPE)

Measured on a 7-point Likert scale (“much better” to “much worse”) [29] at 2 weeks, 3 months and 12 months follow-up. Dichotomized to improved/not improved (improved = much better or better).

#### Activity limitation

Measured by the 23-item RMDQ at 2 weeks, 3 months and 12 months follow-up and converted to a proportional score (0%: no activity limitation, 100%: maximum activity limitation [22]).

#### LBP intensity trajectories

Five LBP trajectories have previously been identified in this cohort by latent class analysis (labelled ‘recovery’, ‘recovery with mild relapses’, ‘slow improvement’, ‘moderate on-going or relapsing’, and ‘severe on-going’) [20]. These were based on weekly measures of LBP intensity (0: no pain, 10: severe pain) collected by SMS for 12 months [30, 31]. To make sure that patients’ individual course matched these trajectories well, only patients with at least 95% posterior probability of belonging to their assigned trajectory were included in the analyses involving this outcome variable. The posterior probabilities were obtained directly from the latent class analysis.

### Data analyses

Preliminary analyses indicated that the QTF categories were differently distributed and differently associated with the baseline characteristics in chiropractic and general practice patients. Consequently, we decided to stratify all analyses according to practice types. Baseline characteristics were reported in each QTF category and significance of differences between the categories was assessed. Continuous variables were summarized using median and 10th and 90th percentiles. For binary, categorical, and ordinal variables proportions were reported with 95% confidence intervals (95% CI). Statistical significance of differences between groups was assessed by the Chi-Square-test for binary and categorical variables and by the Kruskal-Wallis-test for ordinal and continuous variables.

#### Associations between QTF categories and outcomes

The statistical analyses were performed in four steps. 1) Mixed models with clinics as random effects were applied to test for potential clustering within clinics. Because the random effect of clinics did not improve the model fits significantly we ignored such clustering in our further analyses. 2) Crude associations between the QTF categories and the three outcomes were evaluated using linear and logistic regression analyses and statistical significance of differences was assessed at each follow-up time point. The association between the QTF categories and RMDQ scores was illustrated in box plots and distributions within categories described as medians with 95% CI. The association with GPE was described as the proportion (with 95% CI) of patients improved at each time point. The association with the LBP trajectories was described as the proportion (with 95% CI) of patients in each LBP trajectory within each QTF category. To facilitate the interpretation of this analysis, we additionally merged the five LBP trajectories into three trajectory groups of good outcome (‘recovery’ trajectory), intermediate outcome (‘recovery with mild relapses’ + ‘slow improvement’ trajectories) and poor outcome (‘moderate on-going or relapsing’ + ‘severe on-going’ trajectories), which were illustrated in stacked bar charts. We reported the observed difference between the QTF categories in relation to the outcomes and performed a subjective assessment of the relevance of the magnitude. We did not specify limits for when to regard an observed degree of separation as clinically relevant, as we are not aware of any previous work considering a minimal clinically important difference (MCID) to be used when informing patients or clinicians on expected outcomes. Established MCIDs for the RMDQ and pain scales [32, 33] relate to individuals’ change over time and cannot be directly applied for the interpretation of group differences.

Nevertheless they may be kept in mind when judging clinical relevance in our study. The GPE is used in many studies as an anchor to determine MCDIs, so here it is even more obvious that the question of clinical relevant differences in expected outcomes (i.e. probability of experiencing GPE) has to be judged in a different manner. We hence tried to interpret the observed difference in expected outcomes in the sense of obtaining a separation among patients [34], which is large enough to be taken into account in communication with patients and in decision making. 3) Adjusting the linear and logistic regression analyses for socio-demographic factors (age, sex, educational level) we investigated whether observed associations could be explained by the confounding effects of these factors. We did not adjust for factors that may be part of the causal pathway (mediators) between the QTF categories and the outcomes considered. 4) We investigated whether there is an obvious ranking of the four QTF categories on the severity of outcomes by comparing neighbouring categories across all outcomes and follow-up time points:

- ‘LBP alone’ was thus compared to ‘LBP + leg pain above the knee’,
- ‘LBP + leg pain above the knee’ was compared to ‘LBP + leg pain below the knee’
- ‘LBP + leg pain below the knee’ was compared to ‘LBP + NRI’.

A simple count was performed on how many times observed differences between neighbouring categories went in the same direction. For example: did patients with ‘LBP + NRI’ consistently have poorer outcome than patients with ‘LBP + leg pain below the knee’. We excluded the 3 intermediate LBP trajectories (‘recovery with mild relapses’, ‘slow improvement’ and ‘moderate on-going or relapsing’) from this analysis as the order of severity across those trajectories could not be unequivocally determined.

We did two analyses to describe dropout: one comparing those who could be classified using the four QTF categories to those who could not, and one comparing responders at follow-up to non-responders.

For all analyses the significance level was *p* < 0.05. Analyses were performed using STATA 14.

## Results

### Study cohorts

**Fig. 1:**
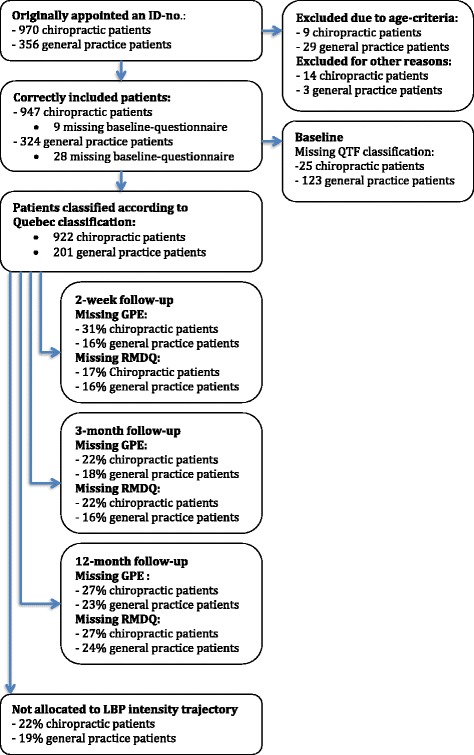
Flow chart from registration to 12 months follow-up

A total of 1271 patients were included, 947 chiropractic patients and 324 patients from general practice (Fig. 1). Each of the chiropractic clinics recruited from 14 to189 patients, 45% of whom were women and the median age was 43 years. General practitioners each included from 1 to 27 patients; 55% were women and the median age was 46 years.

Of the included patients, 97% of chiropractic patients and 62% of patients from general practice could be classified according to the QTF categories. Patients who were not classified according to the QTF categories did not differ significantly on any baseline characteristics from patients who were classified, except for the median age being 1.6 years lower in the non-classified general practice patients (*p* < 0.05) (Table 1a and b).

**Table 1:** Patient-reported baseline characteristics of chiropractic patients (a) and general practice patients (b)

	All chiropractic patients (a) *n* = 947	‘LBP alone’ *n* = 614 (67%)	‘LBP + pain above knee’ *n* = 219 (24%)	‘LBP + pain below knee’ *n* = 69 (7%)	‘LBP + signs of nerve root involvement’ *n* = 20 (2%)	Quebec missing *n* = 25 (3%)	*p*-values^§^
Age in years^a^	43 (28, 59)	42 (27, 58)	45 (30, 60)	47 (32, 63)	48 (33, 66)	37 (23, 62)	<.01
Females^b^	45 (42–48)	42 (38–45)	52 (45–58)	52 (40–64)	55 (32–76)	40 (22–61)	0.03
Physical work load^b^							0.82
- Sitting	23 (21–26)	23 (20–27)	25 (19–31)	22 (13–34)	18 (5–46)	22 (9–45)	
- Sitting/walking	34 (31–37)	34 (30–38)	34 (28–41)	38 (27–52)	41 (19–67)	22 (9–45)	
- Light physical work	21 (19–24)	22 (19–25)	20 (15–26)	17 (9–29)	6 (1–37)	35 (17–57)	
- Heavy physical work	21 (19–24)	21 (18–24)	21 (16–27)	23 (14–36)	35 (15–62)	22 (9–45)	
Sick leave^b^	22 (19–25)	20 (17–24)	26 (20–32)	28 (18–41)	24 (8–52)	14 (4–37)	0.23
Educational level^b^							0.04
- No qualification	9 (7–11)	9 (7–12)	10 (6–15)	6 (2–16)	11 (2–39)	-	
- Vocational training,	26 (23–29)	26 (23–30)	24 (18–30)	32 (22–45)	33 (14–60)	25 (11–47)	
- Higher education < 3 y	16 (13–18)	14 (12–17)	21 (16–27)	8 (3–18)	28 (11–55)	13 (4–34)	
- Higher education 3–4 y	34 (31–38)	33 (30–37)	36 (31–43)	37 (26–50)	28 (11–55)	42 (23–63)	
- Higher education >4 y	15 (13–18)	17 (14–21)	9 (6–14)	17 (9–28)	-	21 (8–43)	
Duration of pain^b^							0.02
- 0–2 weeks	62 (59–66)	66 (62–70)	58 (52–65)	55 (42–66)	39 (18–65)	50 (30–70)	
- 2–4 weeks	14 (12–16)	14 (11–17)	13 (9–18)	11 (5–21)	22 (8–49)	17 (6–39)	
- 1–3 months	10 (9–13)	9 (7–11)	14 (10–20)	12 (6–23)	17 (5–44)	8 (2–30)	
- >3 months	13 (11–16)	11 (9–14)	14 (10–20)	23 (14–35)	22 (8–49)	25 (11–47)	
Previous LBP episodes^b^							0.03
- 0 episodes	16 (14–19)	17 (14–20)	17 (12–22)	6 (2–15)	18 (51–46)	21 (8–43)	
- 1–3 episodes	35 (32–38)	37 (33–41)	33 (27–40)	27 (18–40)	24 (8–52)	29 (14–52)	
- >3 episodes	49 (45–52)	46 (42–50)	50 (44–57)	67 (54–77)	59 (33–81)	50 (30–70)	
LBP last year^b^							<0.001
- ≤30 days	74 (72–77)	79 (76–82)	68 (61–74)	58 (46–70)	53 (27–78)	75 (53–89)	
- >30 days	26 (23–28)	21 (18–24)	32 (26–39)	42 (30–54)	47 (22–73)	25 (11–47)	
LBP intensity^a^	7 (3, 9)	7 (3, 9)	7 (4, 9)	8 (3, 9)	7 (3, 9)	8 (2, 10)	0.07
Leg pain intensity^a^	2 (0, 7)	0 (0, 5)	4 (1, 7)	6 (2, 9)	8 (4, 9)	0 (0, 6)	<0.001
Recovery expectations^a^	9 (4, 10)	10 (4, 10)	9 (4, 10)	8 (2, 10)	9 (1, 10)	10 (4, 10)	<0.001
Depressive symptoms^a^	6 (1, 18)	5 (1, 17)	7.5 (2, 21)	9.5 (2, 21)	6 (2, 34)	7 (2, 19)	<0.001
Fear avoidance beliefs physical activity^a^	13 (6, 20)	13 (6, 20)	12 (5, 19)	14 (2, 20)	12 (5, 20)	14 (6, 22)	0.08
Fear avoidance beliefs work^a^	11 (3, 26)	10 (3, 26)	13 (2, 24)	12 (2, 31)	17.5 (5,31)	11 (4, 33)	0.10
Start Back Tool^b^							<0.001
- Low risk	54 (51–57)	60 (56–64)	41 (34–48)	44 (32–57)	35 (15–62)	46 (26–67)	
- Medium risk	38 (35–41)	34 (30–38)	47 (40–54)	48 (35–60)	41 (19–67)	42 (23–63)	
- High risk	8 (6–10)	6 (4–8)	13 (8–18)	8 (3–18)	24 (8–52)	13 (4–34)	
Activity limitation^a^	52 (17, 83)	52 (13, 83)	56 (26, 83)	57 (17, 83)	74 (17, 90)	59 (15, 85)	0.01
General health^a^	70 (35, 90)	75 (37, 90)	70 (30, 90)	67 (34, 90)	70 (28, 97)	72 (40, 92)	<.01
	All general practice patients (b) *n* = 324	‘LBP alone’ *n* = 97 (48%)	‘LBP + pain above knee’ *n* = 42 (21%)	‘LBP + pain below knee’ *n* = 39 (19%)	‘LBP + signs of nerve root involvement’ *n* = 23 (11%)	Quebec missing *n* = 123 (38%)	*p*-values^§^
Age in years^a^	46 (27–59)	46 (23–61)	47 (31–60)	49 (34–61)	45 (34–60)	44 (26–58)	0.48
Females^b^	55 (49–60)	54 (43–63)	64 (48–78)	46 (31–62)	39 (21–61)	58 (49–67)	0.20
Physical work load^b^							0.45
- Sitting	14 (11–19)	19 (11–29)	6 (1–21)	11 (3–30)	37 (17–62)	18 (11–26)	
- Sitting/walking	32 (27–38)	33 (24–44)	34 (20–52)	36 (20–56)	-	30 (22–39)	
- Light physical work	28 (23–33)	23 (15–34)	23 (11–40)	32 (17–52)	32 (14–57)	31 (22–40)	
- Heavy physical work	25 (21–31)	25 (16–35)	37 (22–55)	21 (9–42)	32 (14–57)	22 (15–31)	
Sick leave^b^	40 (34–46))	34 (25–45)	54 (37–70))	39 (23–58)	56 (31–78)	37 (29–47)	0.12
Educational level^b^							0.47
- No qualification	20 (16–25)	16 (10–26)	26 (14–42)	26 (13–43)	20 (7–45)	19 (13–28)	
- Vocational training,	18 (14–23)	16 (10–26)	23 (12–40)	17 (8–34)	20 (7–45)	18 (12–26)	
- Higher education < 3 y	22 (18–27)	19 (12–29)	26 (14–42)	17 (8–34)	30 (13–55)	23 (16–31)	
- Higher education 3–4 y	29 (24–34)	31 (22–41)	18 (9–34)	34 (20–52)	25 (10–50)	31 (23–40)	
- Higher education >4 y	6 (4–9)	14 (8–23)	3 (0–17)	3 (0–19)	-	3 (1–8)	
Duration of pain^b^							0.03
- 0–2 weeks	39 (33–44)	42 (32–53)	51 (35–67)	20 (9–37)	20 (7–45)	41 (32–50)	
- 2–4 weeks	14 (10–18)	15 (9–25)	5 (1–20)	17 (8–34)	15 (4–40)	14 (8–21)	
- 1–3 months	16 (12–21)	20 (13–30)	8 (2–23)	14 (6–31)	20 (7–45)	15 (10–23)	
- >3 months	32 (27–37)	22 (15–33)	35 (21–52)	49 (32–65)	45 (24–68)	31 (23–40)	
Previous LBP episodes^b^							0.03
- 0 episodes	14 (11–19)	23 (15–34)	8 (2–23)	6 (1–21)	5 (1–32)	14 (9–22)	
- 1–3 episodes	24 (19–29)	16 (10–26)	37 (23–54)	23 (11–40)	25 (10–50)	25 (18–34)	
- >3 episodes	62 (56–67)	60 (50–70)	55 (39–71)	71 (54–84)	70 (45–87)	60 (51–69)	
LBP last year^b^							0.29
- ≤30 days	51 (45–56)	56 (45–66)	53 (36–68)	37 (22–55)	45 (24–68)	51 (42–60)	
- >30 days	49 (44–55)	44 (34–55)	47 (32–64)	63 (45–78)	55 (32–76)	49 (40–58)	
LBP intensity^a^	7 (4, 9)	7 (4, 9)	8 (5, 10)	7 (4, 9)	7 (3, 10)	7 (4, 9)	0.35
Leg pain intensity^a^	3 (0, 9)	0 (0, 6)	5 (0, 9)	7 (3, 9)	6 (4, 10)	3 (0, 9)	<0.001
Recovery expectations^a^	6 (0, 10)	7 (2, 10)	8 (0, 10)	3 (0, 9)	5 (0, 7)	7 (1, 10)	<0.001
Depressive symptoms^a^	9 (2, 26)	8 (2, 22)	7 (1, 27)	15 (2.6, 39)	10 (4, 28)	9 (2, 25)	0.09
Fear avoidance beliefs physical activity^a^	14 (6, 21)	13 (3, 21)	15 (8, 20)	14.5 (5, 22)	17 (9, 24)	13.5 (6, 21)	0.01
Fear avoidance beliefs work^a^	14 (3, 28)	11 (3, 27)	18 (6, 27)	16 (4, 30)	23 (12, 38)	14 (3, 29)	0.001
Start Back Tool^b^							<0.001
- Low risk	41 (36–47)	51 (40–62)	31 (17–50)	25 (13–44)	27 (9–57)	44 (35–54)	
- Medium risk	36 (30–41)	28 (19–39)	59 (41–75)	25 (13–44)	33 (13–63)	38 (29–47)	
- High risk	23 (18–28)	21 (14–32)	9 (3–27)	50 32–68)	40 17–68)	18 (12–26)	
Activity limitation^a^	61 (22, 87)	54 (17, 83)	61 (35, 87)	74 (14, 96)	72 (31, 91)	65 (22, 87)	<0.001
General health^a^	60 (26, 90)	65 (35, 90)	60 (19, 90)	50 (20, 86)	50 (16, 74)	65 (25, 90)	0.005

*LBP* low back pain

^a^Median (10%, 90% centiles)

^b^Proportion (95% CI)

^§^ *P*-values for test of any differences across the four QTF categories

At the 1-year follow-up, 73 and 76% responded to questionnaires in chiropractic and general practice respectively. The dropout rates did not differ between the four QTF categories. In chiropractic practice, non-responders were on average 6 years younger and were more often male (45% vs. 39%), had slightly lower recovery expectations, slightly more depressive symptoms and marginally higher fear avoidance beliefs than responders (*p* < 0.05). General practice non-responders were more often male (60% vs. 38%) and more of them had heavy physical work (38% vs. 23%) as compared to responders (*p* < 0.05). On all other baseline characteristics, non-responders did not differ significantly from responders.

In total, 27% of the chiropractic patients and 38% from general practice could not be allocated to a LBP intensity trajectory with at least 95% probability. General practice patients with no assigned LBP intensity trajectory were marginally less depressive (*p* < 0.05) and were less frequently in the SBT high-risk group (*p* < 0.01). There were no other statistically significant differences between patients with and without an assigned trajectory in the two cohorts.

### Baseline characteristics of the four QTF categories (objective 1)

The majority of chiropractic patients presented with ‘LBP alone’ (67%) and only 2% presented with ‘LBP + NRI’ whereas 48% of patients in general practice presented with ‘LBP alone’ and 11% with ‘LBP + NRI’. In both settings, the majority of the 17 baseline characteristics differed significantly between the four QTF categories with effects in the same direction and of similar magnitude (Table 1a and b). Generally, those with ‘LBP alone’ had the least severe profile and patients with ‘LBP + NRI’ were most severely affected on the largest number of parameters.

### Crude associations between QTF categories and outcomes (objective 2)

#### Activity limitation

**Fig. 2:**
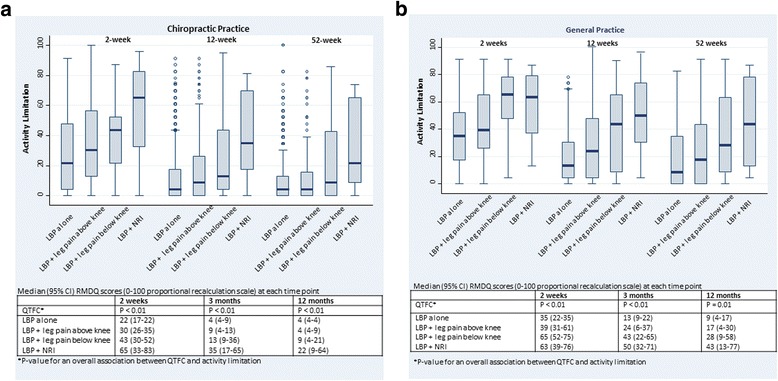
Median RMDQ scores and the distribution of the scores in the four QTF categories in 947 chiropractic patients (**a**) and 324 patients from general practice (**b**) at 2 weeks, 3 months, and 12 months follow-up

Statistically significant associations between the QTF categories and activity limitation were present at all follow-up time points in both cohorts (Fig. 2a and b). Generally, ‘LBP alone’ had the least activity limitation at all time points and ‘LBP + NRI’ had the most activity limitation. Differences were substantial with for example an expected median score of 35/100 after 3 months for patients with ‘LBP + NRI’ compared to an expected score of 13/100 in patients with ‘LBP + leg pain below the knee’ and 4/100 for patients with ‘LBP alone’ in chiropractic practice (tables in the lower part of Fig. 2a and b). However, activity limitation scores varied within each QTF category, implying that some individuals would experience outcomes that differed substantially from the mean score of their QTF category.

### Global perceived effect

In both settings, there was a statistically significant association between the QTF categories and GPE at 2 weeks follow-up with the largest proportion of improved patients in the ‘LBP alone’ and the ‘LBP + leg pain above the knee’ categories (Table 2). A larger proportion of patients with ‘LBP + leg pain above the knee’ compared to patients with ‘LBP + leg pain below the knee’ improved in both cohorts and in chiropractic practice the probability of being improved further decreased considerably for patients with ‘LBP + NRI’. There were no statistically significant associations at the later follow-up time points.

**Table 2:** Relationship between QTF categories and global perceived effect (GPE)

QTF categories	2 weeks, proportion improved % (95% CI)		3 months, proportion improved % (95% CI)		12 months, proportion improved % (95% CI)	
	Chiropractic patients *P* < 0.01*	General practice patients *P* < 0.01*	Chiropractic patients *P* = 0.2*	General practice patients *P* = 0.2*	Chiropractic patients *P* = 0.4*	General practice patients *P* = 0.5*
All patients	74 (70–77)	36 (31–42)	82 (79–85)	60 (54–66)	73 (23–30)	54 (48–60)
‘LBP alone’	77 (73–81)	49 (38–60)	82 (78–85)	69 (58–78)	74 (69–78)	53 (42–65)
‘LBP + leg pain above the knee’	72 (64–79)	43 (28–60)	85 (79–90)	66 (49–80)	75 (67–81)	66 (48–80)
‘LBP + leg pain below the knee’	61 (46–74)	19 (9–38)	73 (59–83)	46 (29–65)	63 (49–76)	54 (34–72)
‘LBP + NRI’	40 (17–68)	20 (7–45)	87 (55–97)	56 (31–78)	73 (43–91)	44 (20–70)

Proportions improved after 2 weeks, 3 months and 12 months

*LBP* low back pain, *NRI* nerve root involvement

*P-value for an overall association between QTF categories and GPE

### LBP intensity trajectories

**Fig. 3:**
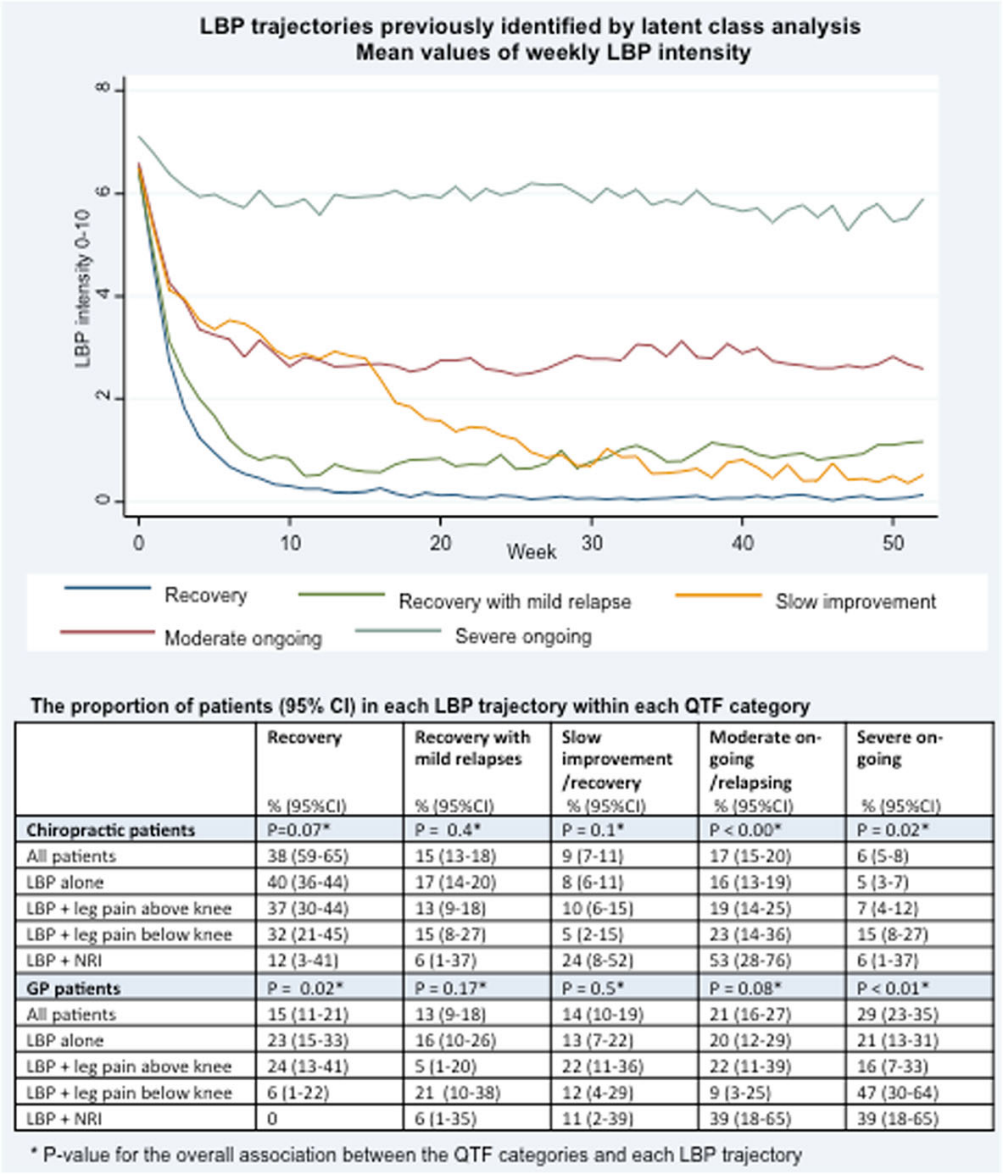
Mean low back pain intensity over one year in the five trajectory groups (*upper part*); the statistical significance of the association with QTF classification; and the proportion of patients assigned to each of the five trajectories within the four QTF

The QTF categories were statistically significantly or borderline significantly associated with the ‘recovery’, ‘moderate on-going’ and ‘severe on-going trajectories’ in both settings. The table in the lower part of Fig. 3 shows the distribution of the five trajectories within each QTF category. In the chiropractic cohort, the majority of patients were in the ‘recovery’ trajectory (38%) and only 6% had ‘severe on-going pain’, whereas only 15% of general practice patients were in the ‘recovery’ trajectory and 29% had ‘severe on-going pain’. The upper part of Fig. 3 shows the mean LBP intensity in each week within the five trajectories.

**Fig. 4:**
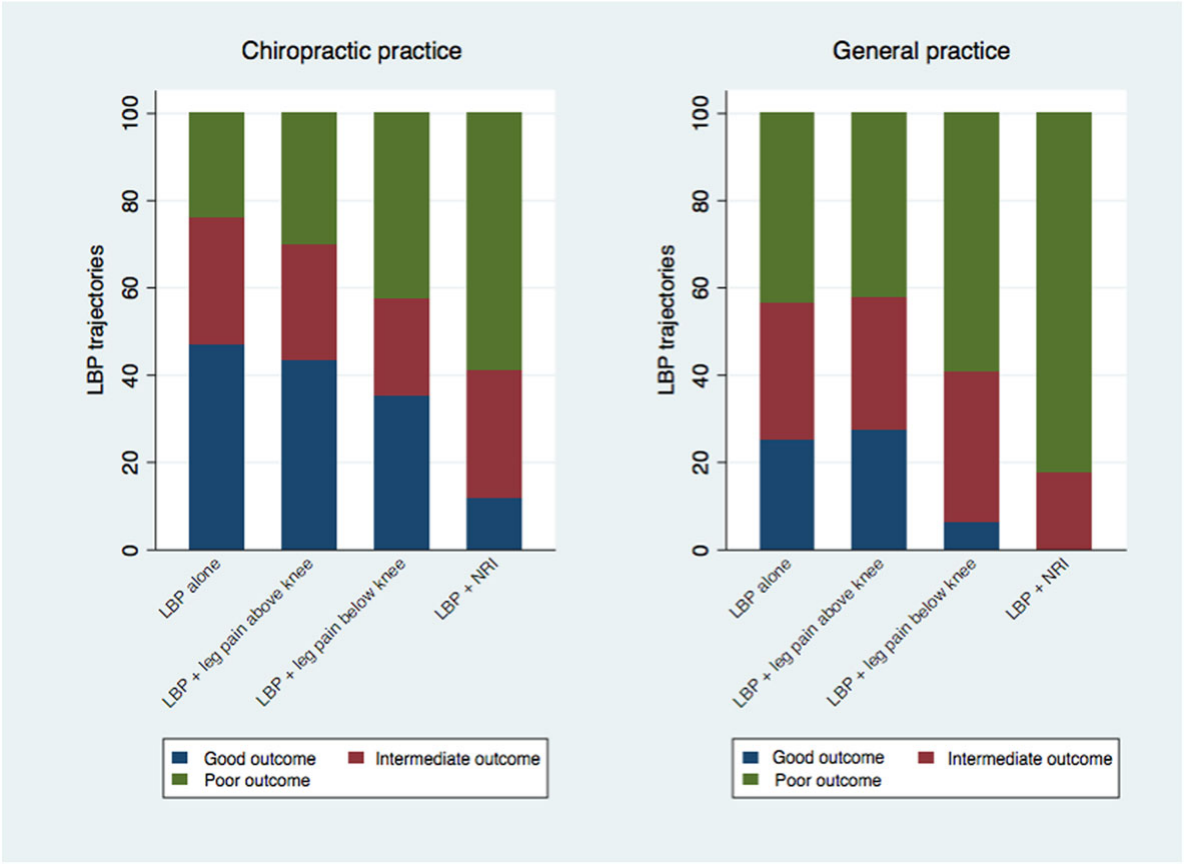
Distribution of the three low back pain trajectories groups (good, intermediate, and poor outcome) within the four QTF categories. Based on 947 chiropractic patients and 324 patients from general practice

The distribution across the five trajectories was similar for patients with ‘LBP alone’ and patients with ‘LBP + leg pain above the knee’ in both cohorts, whereas for patients with ‘LBP + leg pain below the knee’ and in particular for patients with ‘LBP + NRI’ we observed a larger proportion of patient in the trajectories with on-going pain. The ranking across the QTF categories became even more visible when merging the five LBP trajectories into three trajectory groups of good, intermediate, and poor outcome (Fig. 4). In chiropractic practice there was a decreasing proportion of patients in the good outcome trajectory and an increasing proportion of patients in the poor outcome trajectory going from ‘LBP alone’, across ‘LBP + leg pain above the knee’ and ‘LBP + leg pain below the knee’, to ‘LBP + NRI’. In general practice patients with ‘LBP alone’ and patients with ‘LBP + leg pain above the knee’ had similar distributions across the three trajectory groups, whereas the proportion of patients in the poor outcome trajectory was substantially larger in patients with ‘LBP + leg pain below the knee’ and even more so for patients with ‘LBP + NRI’.

### Adjusted associations between QTF categories and outcomes (objective 3)

Adjusting simultaneously for age, sex and educational level did not change the statistical significance of the association between the QTF categories and outcomes except for the association with the recovery trajectory, which after adjusting was no longer statistically significant. The estimates did not change to a relevant degree (Additional file 1). The largest change in odds ratio occurred in the association between ‘LBP + NRI’ and the poor outcome trajectory, where the odds ratio changed from 5.53 to 4.27 in the chiropractic cohort. In the case of significant associations, the ordering of the effect estimates across the four categories did not change when adjusting.

### Ranking of the four QTF categories (objective 4)

**Fig. 5:**
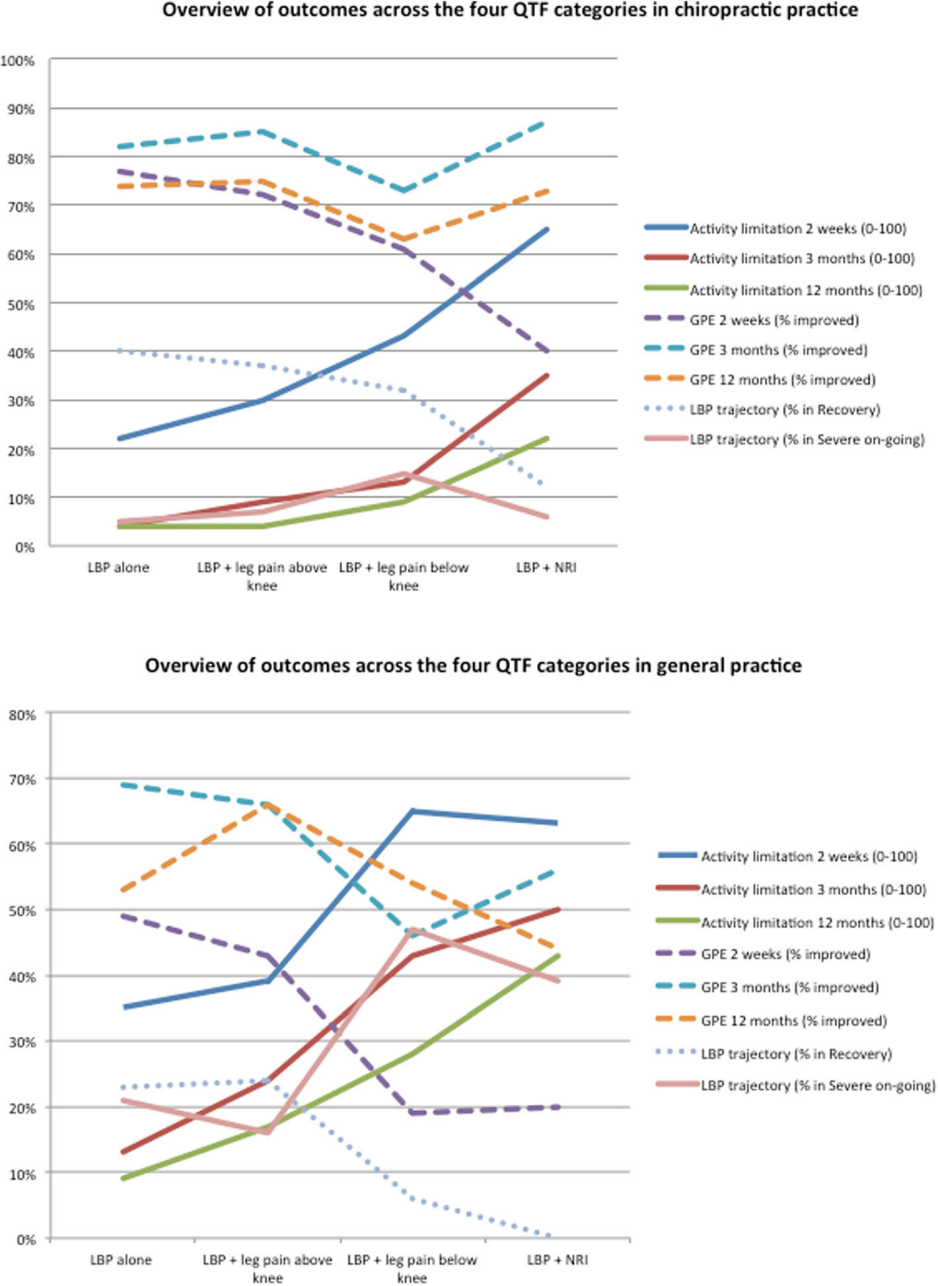
Trends across the four QTF categories on all outcomes at each follow-up time point. *Solid lines* represent outcomes where an *increase* indicate poorer outcome. *Dashed lines* represent outcomes where a *decrease* indicate poorer outcome

For nearly all outcomes with a statistically significant association with the QTF categories a ranking of increasing severity from the QTF category 1 to 4 could be observed although for some outcomes it was difficult to differentiate between ‘LBP alone’ and ‘LBP + leg pain above the knee’ and for others ‘LBP + leg pain below the knee’ was very similar to ‘LBP + NRI’ (Fig. 5). In the comparisons of the outcomes of neighbouring QTF categories, ‘LBP + leg pain above the knee’ had worse outcomes than ‘LBP alone’ in 11 out of 16 possible comparisons, ‘LBP + leg pain below the knee’ was associated with worse outcomes than ‘LBP + leg pain above the knee’ in 15 out of 16 comparisons, and ‘LBP + NRI’ had worse outcomes than ‘LBP + leg pain below the knee’ in 10 of 16 comparisons.

## Discussion

### Summary of main findings

To our knowledge, this is the first study in primary care in which the presence and extent of leg pain and NRI were determined by clinical assessment and the separative capacity of the four QTF categories was examined. We found that patients in the four QTF categories had different clinical presentations when seeking care, and that they also differed on outcomes. Pain and activity limitation were worse at all follow-up time points for patients with leg pain compared with patients with ‘LBP alone’, especially so for patients with pain below the knee and NRI. Importantly, our results suggest that patients with ‘LBP + NRI’ constitute a subgroup with its own characteristics and course and that the clinical examination thus is an important part of differentiating between categories of LBP-related leg pain. We also confirmed that in epidemiological studies when a clinical examination is not feasible the differentiation between pain above and below the knee does carry valuable information. The observed ranking of increasing severity in outcomes from the QTF category 1 to category 4 was similar for patients seen in chiropractic and general practice in spite of the fact that the type of care is not the same in chiropractic and general practice.

Consistent group differences demonstrated that the QTF categories do identify distinct LBP subgroups. The differences in expected outcomes between ‘LBP alone’ and ‘LBP + NRI’ in both cohorts and between ‘LBP + leg pain below the knee’ and ‘LBP + NRI’ in the chiropractic cohort reached a magnitude we regard as clinically relevant, i.e. provided a clinically relevant degree of separation [34]. Thus, the QTF classification is an easily applicable tool providing insight about the expected outcome of patients at a group level and it may be useful in the communication with patients. However, the variation within categories also implied that the QTF classification is not an accurate predictor of individuals’ outcomes.

An earlier analysis of the same cohorts has shown that the patients in general practice were generally worse on a wide range of health parameters than the chiropractic patients at presentation [19]. The present study furthermore demonstrated that a considerably smaller proportion of chiropractic patients than general practice patients were categorized as having ‘LBP + NRI’. This may be caused by symptoms of NRI triggering different care seeking behaviors than other back pain complaints, but might also be due to the collection of information on signs of NRI. According to the study protocol, the chiropractors had to do a thorough neurological examination whereas general practitioners were to answer a yes/no question on the presence of abnormal neurological findings. Thus, in chiropractic practice the classification of category 4 followed a standardized examination that may have resulted in a more precise diagnosis compared with general practice. This may in turn explain why the biggest difference between any of the four QTF categories in chiropractic patients was between patients with ‘LBP + leg pain below the knee’ and patients with ‘LBP + NRI’ whereas the difference between these two categories in general practice was less pronounced.

### Comparison with existing literature

The results showing that LBP with leg pain below the knee with or without NRI is associated with a worse prognosis than LBP alone or LBP with leg pain above the knee is in line with previous studies from primary and secondary care in which absolute disability scores were about three times higher in patients having LBP with leg pain below the knee and 4–8 times higher in patients with LBP with NRI compared to patients with LBP alone at both 3-months and 1-year follow-up [8, 9, 13, 15]. In contrast to one of these studies [13], we observed different outcomes for patients with LBP with leg pain above the knee as compared to patients with LBP with leg pain below the knee. This difference between the two studies may be caused by the previous study investigating LBP of very long duration in which pain location may become a less important element in a complex condition.

### Strengths and limitations

A major strength of this study was the standardized clinical examination for signs of NRI conducted in the chiropractic cohort. Also the sample size was adequately large to result in relative precise estimates of group differences despite the limitation of some infrequent QTF categories. Data were collected prospectively and included both patient-reported information and clinical data, and to our knowledge this was the first study to include LBP trajectories derived from latent class analyses as an outcome measure.

LBP is a largely episodic/recurrent condition for most individuals manifesting itself on and off over the entire lifespan [35–37]. To establish a more detailed description of pain patterns, data collection by means of frequent text messaging on mobile phones has been introduced and different back pain trajectories have been identified and linked to clinical parameters in several other studies [38–41]. We used LBP trajectories as an outcome measure although the psychometric properties of this outcome measure have not been investigated. However these LBP trajectories represent patterns of LBP similar to those observed in other primary care cohorts [39, 42–44] and provided an opportunity to include a measure of the clinical course that does not assume that pain outcomes differ only in severity at certain time points but also in course pattern. It was reassuring that the general findings on this outcome variable reflected the same relationships as those on activity limitation.

Obviously, the study is limited by a lack of standardization of the clinical examination in general practice which may have caused less distinct differences between leg pain below the knee and NRI in that cohort than what is truly the case. Another limitation of the study was the incomplete follow-up in both cohorts and the fact that only two thirds of patients from general practice were classified according to the QTF categories. However, patients classified were similar to patients not classified and responders were similar to non-responders. It is therefore unlikely that the results are influenced by this incompleteness. Also, it is a limitation that pain location was not obtained from patients categorized with ‘LBP + NRI’. It would be useful to know what proportion of this group actually reports pain below the knee.

The only outcome that was not associated with the QTF categories was GPE at the 3-months and 1-year follow-ups. This may imply that GPE is likely to be unsuitable as a long-term outcome. It has been suggested that patients have difficulty taking their baseline status into account when scoring the GPE, and that the GPE ratings are influenced by peoples current health status [29], which is likely to become increasingly problematic with longer follow-up periods.

It was outside the scope of this study to investigate the value of the QTF categories in comparison to or in addition to other classification tools or prognostic markers.

### Implications for clinical practice and future research

This study demonstrated that the QTF categories provide a simple way for clinicians to classify patients with non-specific LBP into subgroups with expected different outcomes. The results from this study underpin the importance of establishing a diagnosis of NRI based on a clinical examination and not merely on self-reported symptoms and our results support that both localization of pain and NRI are relevant prognostic factors [9, 13, 15, 16]. Next it should be investigated if the QTF categories add information to existing LBP prediction models and if the QTF categories moderate the effect of recommended treatments for LBP. Generally, in the design of research projects it should be recognized that data from the clinical examination are needed when identification of relatively homogeneous groups of patients with LBP-related leg pain is required. Finally, the results imply that systematic reviews and meta-analyses should assess the consequences of combining leg pain categories and whenever possible report the different categories separately.

## Conclusion

Our results confirm that the QTF categories do identify LBP subgroups differing in baseline characteristics as well as in expected outcomes. The latter appear to be of a clinically relevant degree and reflect a ranking in severity. Patient outcomes are best when pain is restricted to the low back, worse if the pain radiates down the leg (worst with pain below the knee), and most severe if there are neurological symptoms in the leg as well. To identify the latter group, a clinical examination is warranted. Clearly, the four QTF categories deserve more interest in relation to research into improvement of prediction tools and treatment decisions.

## Additional file

Additional file 1: Unadjusted and adjusted^#^ odds ratios (OR) for the comparison of the four Quebec Task Force categories (QTFC) and *p*-values for the effect of QTFC on outcomes before and after adjustment in the two cohorts. (DOCX 20 kb)

## Abbreviations

FABQ: Fear-avoidance beliefs questionnaire
GPE: Global perceived effect
LBP: Low back pain
NRI: Nerve root involvement
NRS: Numeric rating scale
QTF: Quebec Task Force
QTFC: Quebec Task Force categories
RMDQ: Roland Morris Disability Questionnaire

## References

[1] Vos T, Flaxman AD, Naghavi M, Lozano R, Michaud C, Ezzati M, Shibuya K, Salomon JA, Abdalla S, Aboyans V (2012). Years lived with disability (YLDs) for 1160 sequelae of 289 diseases and injuries 1990–2010: a systematic analysis for the Global Burden of Disease Study 2010. Lancet.

[2] United States Bone and Joint Initiative. The Burden of Musculoskeletal Diseases in the United States (BMUS) TE, 2014. Rosemont, IL. Available at http://www.boneandjointburden.org. Accessed 3 May 2016.

[3] Flachs EM EL, Koch MB, Ryd JT, Dibba E, Skov-Ettrup L, Juel K (2015). Sygdomsbyrden i Danmark – sygdomme. Statens Institut for Folkesundhed, Syddansk Universitet.

[4] van Tulder M, Becker A, Bekkering T, Breen A, del Real MT, Hutchinson A, Koes B, Laerum E, Malmivaara A (2006). Chapter 3. European guidelines for the management of acute nonspecific low back pain in primary care. Eur Spine J.

[5] Karayannis NV, Jull GA, Hodges PW (2012). Physiotherapy movement based classification approaches to low back pain: comparison of subgroups through review and developer/expert survey. BMC Musculoskelet Disord.

[6] Fairbank J, Gwilym SE, France JC, Daffner SD, Dettori J, Hermsmeyer J, Andersson G (2011). The role of classification of chronic low back pain. Spine.

[7] O SW. Scientific approach to the assessment and management of activity-related spinal disorders. A monograph for clinicians. Report of the Quebec Task Force on Spinal Disorders. Spine. 1987;12(7 Suppl):S1-59. 2961086

[8] Atlas SJ, Deyo RA, Patrick DL, Convery K, Keller RB, Singer DE (1996). The Quebec Task Force classification for spinal disorders and the severity, treatment, and outcomes of sciatica and lumbar spinal stenosis. Spine.

[9] Loisel P, Vachon B, Lemaire J, Durand MJ, Poitras S, Stock S, Tremblay C (2002). Discriminative and predictive validity assessment of the quebec task force classification. Spine.

[10] Werneke MW, Hart DL (2004). Categorizing patients with occupational low back pain by use of the Quebec Task Force Classification system versus pain pattern classification procedures: discriminant and predictive validity. Phys Ther.

[11] Selim AJ, Ren XS, Fincke G, Deyo RA, Rogers W, Miller D, Linzer M, Kazis L (1998). The importance of radiating leg pain in assessing health outcomes among patients with low back pain. Results from the Veterans Health Study. Spine.

[12] Kongsted A, Kent P, Albert H, Jensen TS, Manniche C (2012). Patients with low back pain differ from those who also have leg pain or signs of nerve root involvement - a cross-sectional study. BMC Musculoskelet Disord.

[13] Kongsted A, Kent P, Jensen TS, Albert H, Manniche C (2013). Prognostic implications of the Quebec Task Force classification of back-related leg pain: an analysis of longitudinal routine clinical data. BMC Musculoskelet Disord.

[14] Hider SL, Whitehurst DG, Thomas E, Foster NE (2015). Pain location matters: the impact of leg pain on health care use, work disability and quality of life in patients with low back pain. Eur Spine J.

[15] Hill JC, Konstantinou K, Egbewale BE, Dunn KM, Lewis M, van der Windt D (2011). Clinical outcomes among low back pain consulters with referred leg pain in primary care. Spine.

[16] Konstantinou K, Hider SL, Jordan JL, Lewis M, Dunn KM, Hay EM (2013). The impact of low back-related leg pain on outcomes as compared with low back pain alone: a systematic review of the literature. Clin J Pain.

[17] Ashworth J, Konstantinou K, Dunn KM (2011). Prognostic factors in non-surgically treated sciatica: a systematic review. BMC Musculoskelet Disord.

[18] Koes BW, van Tulder M, Lin CW, Macedo LG, McAuley J, Maher C (2010). An updated overview of clinical guidelines for the management of non-specific low back pain in primary care. Eur Spine J.

[19] Hestbaek L, Munck A, Hartvigsen L, Jarbol DE, Sondergaard J, Kongsted A (2014). Low back pain in primary care: a description of 1250 patients with low back pain in danish general and chiropractic practice. Int J Fam Med.

[20] Kongsted A, Kent P, Hestbaek L, Vach W (2015). Patients with low back pain had distinct clinical course patterns that were typically neither complete recovery nor constant pain. A latent class analysis of longitudinal data. Spine J.

[21] Eirikstoft H, Kongsted A (2014). Patient characteristics in low back pain subgroups based on an existing classification system. A descriptive cohort study in chiropractic practice. Man Ther.

[22] Kent P, Lauridsen HH (2011). Managing missing scores on the Roland Morris Disability Questionnaire. Spine.

[23] Jensen MP, Miller L, Fisher LD (1998). Assessment of pain during medical procedures: a comparison of three scales. Clin J Pain.

[24] Kongsted A, Vach W, Axo M, Bech RN, Hestbaek L (2014). Expectation of recovery from low back pain: a longitudinal cohort study investigating patient characteristics related to expectations and the association between expectations and 3-month outcome. Spine.

[25] Bech P, Rasmussen NA, Olsen LR, Noerholm V, Abildgaard W (2001). The sensitivity and specificity of the Major Depression Inventory, using the Present State Examination as the index of diagnostic validity. J Affect Disord.

[26] Waddell G, Newton M, Henderson I, Somerville D, Main CJ (1993). A Fear-Avoidance Beliefs Questionnaire (FABQ) and the role of fear-avoidance beliefs in chronic low back pain and disability. Pain.

[27] Rabin R, de Charro F (2001). EQ-5D: a measure of health status from the EuroQol Group. Ann Med.

[28] Hill JC, Dunn KM, Lewis M, Mullis R, Main CJ, Foster NE, Hay EM (2008). A primary care back pain screening tool: identifying patient subgroups for initial treatment. Arthritis Rheum.

[29] Kamper SJ, Ostelo RW, Knol DL, Maher CG, de Vet HC, Hancock MJ (2010). Global Perceived Effect scales provided reliable assessments of health transition in people with musculoskeletal disorders, but ratings are strongly influenced by current status. J Clin Epidemiol.

[30] Axen I, Bodin L, Bergstrom G, Halasz L, Lange F, Lovgren PW, Rosenbaum A, Leboeuf-Yde C, Jensen I (2012). The use of weekly text messaging over 6 months was a feasible method for monitoring the clinical course of low back pain in patients seeking chiropractic care. J Clin Epidemiol.

[31] Kongsted A, Kent P, Axen I, Downie AS, Dunn KM (2016). What have we learned from ten years of trajectory research in low back pain?. BMC Musculoskelet Disord.

[32] Bombardier C, Hayden J, Beaton DE (2001). Minimal clinically important difference. Low back pain: outcome measures. J Rheumatol.

[33] Ostelo RW, Deyo RA, Stratford P, Waddell G, Croft P, Von Korff M, Bouter LM, de Vet HC (2008). Interpreting change scores for pain and functional status in low back pain: towards international consensus regarding minimal important change. Spine.

[34] Royston PSW (2004). A new measure of prognostic separation in survival data. Stat Med.

[35] Axen I, Leboeuf-Yde C (2013). Trajectories of low back pain. Best Pract Res Clin Rheumatol.

[36] Dunn KM, Hestbaek L, Cassidy JD (2013). Low back pain across the life course. Best Pract Res Clin Rheumatol.

[37] Lemeunier N, Leboeuf-Yde C, Gagey O (2012). The natural course of low back pain: a systematic critical literature review. Chiropr Man Ther.

[38] Leboeuf-Yde C, Lemeunier N, Wedderkopp N, Kjaer P (2013). Evidence-based classification of low back pain in the general population: one-year data collected with SMS Track. Chiropr Man Ther.

[39] Axen I, Bodin L, Bergstrom G, Halasz L, Lange F, Lovgren PW, Rosenbaum A, Leboeuf-Yde C, Jensen I (2011). Clustering patients on the basis of their individual course of low back pain over a six month period. BMC Musculoskelet Disord.

[40] Kongsted A, Leboeuf-Yde C (2009). The Nordic back pain subpopulation program--individual patterns of low back pain established by means of text messaging: a longitudinal pilot study. Chiropr Osteopath.

[41] Macedo LG, Maher CG, Latimer J, McAuley JH, Hodges PW, Rogers WT (2014). Nature and determinants of the course of chronic low back pain over a 12-month period: a cluster analysis. Phys Ther.

[42] Dunn KM, Jordan K, Croft PR (2006). Characterizing the course of low back pain: a latent class analysis. Am J Epidemiol.

[43] Downie AS, Hancock MJ, Rzewuska M, Williams CM, Lin CW, Maher CG (2016). Trajectories of acute low back pain: a latent class growth analysis. Pain.

[44] Deyo RA, Bryan M, Comstock BA, Turner JA, Heagerty P, Friedly J, Avins AL, Nedeljkovic SS, Nerenz DR, Jarvik JG (2015). Trajectories of symptoms and function in older adults with low back disorders. Spine.

