# Supplementary material for: Leg pain location and neurological signs relate to outcomes in primary care patients with low back pain
Source: BMC Musculoskelet Disord. 2017 Mar 31;18:133. doi: 10.1186/s12891-017-1495-3 (PMC5374567; doi:10.1186/s12891-017-1495-3)
Supplement: Additional file 1: — Unadjusted and adjusted# odds ratios (OR) for the comparison of the four Quebec Task Force categories (QTFC) and p-values for the effect of QTFC on outcomes before and after adjustment in the two cohorts. (DOCX 20 kb) [file 12891_2017_1495_MOESM1_ESM.docx]

Additional file 1 Unadjusted and adjusted^#^ odds ratios (OR) for the comparison of the four Quebec Task Force categories (QTFC) and p-values for the effect of QTFC on outcomes before and after adjustment in the two cohorts

| **Chiropractic practice** | **Global perceived effect 2 weeks** | | **Global perceived effect 3 months** | | **Global perceived effect 12 months** | |
| --- | --- | --- | --- | --- | --- | --- |
|  | Unadjusted OR | Adjusted OR | Unadjusted OR | Adjusted OR | Unadjusted OR | Adjusted OR |
| **QTFC** | P < 0.01 | P <0.01 | P =0.22 | P = 0.20 | P = 0.44 | P = 0.48 |
| **LBP alone**  **LBP + Leg pain above knee**  **LBP + leg pain below knee**  **LBP + NRI** | 1  0.74 (0.49-1.14)  0.45 (0.24-0.86)  0.19 0.07-0.56) | 1  0.70 (0.45-1.08)  0.42 (0.22-0.82)  0.20 (0.07-0.61) | 1  1.25 (0.77-2.03)  0.59 (0.31-1.11)  1.43 (0.32-6.46) | 1  1.21 (0.73-1.99)  0.57 (0.29-1.10)  2.44 (0.31-19.17) | 1  1.05 (0.70-1.59)  0.61 (0.33-1.14)  0.98 (0.31-3.14) | 1  1.11 (0.73-1.70)  0.67 (0.35-1.28)  1.57 (0.42-5.80) |

| **General practice** | **Global perceived effect 2 weeks** | | **Global perceived effect 3 months** | | **Global perceived effect 12 months** | |
| --- | --- | --- | --- | --- | --- | --- |
|  | Unadjusted OR | Adjusted OR | Unadjusted OR | Adjusted OR | Unadjusted OR | Adjusted OR |
| **QTFC** | P < 0.01 | P = 0.02 | P = 0.15 | P = 0.26 | P = 0.47 | P = 0.51 |
| **LBP alone**  **LBP + leg pain above knee**  **LBP + leg pain below knee**  **LBP + NRI** | 1  0.78 (0.36-1.71)  0.25 (0.09-0.66)  0.26 (0.08-0.83) | 1  0.68 (0.29-1.60)  0.25 (0.09-0.69)  0.28 (0.08-0.95) | 1  0.86 (0.38-1.95)  0.39 (0.17-0.92)  0.56 (0.20-1.58) | 1  1.03 (0.45-2.49)  0.44 (0.18-1.07)  0.62 (0.21-1.82) | 1  1.68 (0.73-3.86)  1.01 (0.42-2.41)  0.68 (0.23-2.02) | 1  1.80 (0.73-4.40)  1.08 (0.43-2.68)  0.77 (0.25-2.39) |

| **Chiropractic practice** | **Activity limitation 2 weeks** | | **Activity limitation 3 months** | | **Activity limitation 12 months** | |
| --- | --- | --- | --- | --- | --- | --- |
|  | Unadjusted  Beta-coefficients | Adjusted  Beta-coefficients | Unadjusted  Beta-coefficients | Adjusted  Beta-coefficients | Unadjusted  Beta-coefficients | Adjusted  Beta-coefficients |
| **QTFC** | P < 0.01 | P < 0.01 | P < 0.01 | P < 0.01 | P < 0.01 | P < 0.01 |
| **LBP alone**  **LBP + leg pain above knee**  **LBP + leg pain below knee**  **LBP + NRI** | 0  8.70 (1.68-15.71)  21.74 (10.05-33.43)  39.13 (18.56-59.70) | 0  7.51(1.16-13.86)  16.66 (6.03-27.29)  37.31(18.42-56.21) | 0  4.35 (0.26-8.44))  8.70(2.17-15.22)  30.43 (18.42-42.45) | 0  0 (-3.74-3.74)  13.04 (7.06-19.02)  26.09 (14.91-37.26) | 0  0 (-3.55-3.55)  4.35 (-1.45-10.14)  17.39 (7.28-27.51) | 0  -0.20 (-2.77-2.37)  3.61 (-0.63-7.86)  15.36 (7.88-22.84) |

| **General practice** | **Activity limitation 2 weeks** | | **Activity limitation 3 months** | | **Activity limitation 12 months** | |
| --- | --- | --- | --- | --- | --- | --- |
|  | Unadjusted  Beta-coefficients | Adjusted  Beta-coefficients | Unadjusted  Beta-coefficients | Adjusted  Beta-coefficients | Unadjusted  Beta-coefficients | Adjusted  Beta-coefficients |
| **QTFC** | P < 0.01 | P < 0.01 | P < 0.01 | P < 0.01 | P = 0.02 | P < 0.01 |
| **LBP alone**  **LBP + leg pain above knee**  **LBP + leg pain below knee**  **LBP + NRI** | 0  4.35 (-10.86-19.55)  30.43 (14.26-46.61)  26.09 (6.97-45.21) | 0  8.48 (-6.76-22.72)  26.42(11.60-41.25)  21.57 (4.09-39.05) | 0  13.04 (-3.16-29.25)  30.43 (12.81-48.06)  39.13 (17.61-60.64) | 0  7.60 (-7.70-22.89)  22.90 (6.72-39.09)  34.53(14.80-5 4.25) | 0  8.70 (-9.24-26.63)  21.74 (2.35-41.13)  34.78 (10.03-59.53) | 0  4.35 (-14.35-23.05)  21.74 (2.09-41.39)  43.48 (18.35-68.61) |

| **Chiropractic practice** | **‘Recovery’*** | | **‘Recovery with mild relapses’*** | | **‘Slow improvement/recovery’*** | | **‘Moderate on-going/relapsing’*** | | **‘Severe on-going’*** | |
| --- | --- | --- | --- | --- | --- | --- | --- | --- | --- | --- |
|  | Unadjusted OR | Adjusted OR | Unadjusted OR | Adjusted OR | Unadjusted OR | Adjusted OR | Unadjusted OR | Adjusted OR | Unadjusted OR | Adjusted OR |
|  | P = 0.07 | P=0.18 | P = 0.4 | P = 0.35 | P = 0.17 | P = 0.06 | P < 0.00 | P = 0.01 | P = 0.02 | P = 0.01 |
| **Local LBP alone**  **LBP + leg pain**  **above knee**  **LBP + leg pain**  **below knee**  **LBP + NRI** | 1.0  0.88 (0.63-1.22)  0.70 (0.40-1.24)  0.20 (0.05-0.89) | 1.0  0.91 (0.64-1.27)  0.70 ( 0.38-1.27)  0.23 (0.05-1.05) | 1.0  0.72 (0.45-1.16)  0.89 (0.42-1.86)  0.31 (0.04-2.40) | 1.0  0.68 (0.42-1.11)  0.88 ( 0.41-1.88)  0.33 (0.04-2.59) | 1.0  1.21 (0.69-2.12)  0.60 (0.18-2.00)  3.52 (1.10-11.25) | 1.0  1.29 ( 0.73-2.28)  0.66 (0.20-2.20)  4.85 (1.43-16.43) | 1.0  1.24 (0.81-1.90)  1.65 (0.87-3.14)  6.12 (2.30-16.29) | 1.0  1.14 (0.74-1.77)  1.54 (0.79-3.01)  5.71 (1.98-16.45) | 1.0  1.44 (0.74-2.79)  3.35 (1.50-7.49)  1.19 (0.15-9.28) | 1.0  1.59 (0.81-3.14)  3.88 (1.69-8.92)  1 |

| **General practice** | **‘Recovery’*** | | **‘Recovery with mild relapses’*** | | **‘Slow improvement/recovery’*** | | **‘Moderate on-going/relapsing’*** | | **‘Severe on-going’*** | |
| --- | --- | --- | --- | --- | --- | --- | --- | --- | --- | --- |
|  | Unadjusted OR | Adjusted OR | Unadjusted OR | Adjusted OR | Unadjusted OR | Adjusted OR | Unadjusted OR | Adjusted OR | Unadjusted OR | Adjusted OR |
|  | P = 0.02 | P = 0.13 | P = 0.17 | P = 0.20 | P = 0.5 | P =0.24 | P = 0.08 | P = 0.07 | P < 0.01 | P = 0.02 |
| **Local LBP alone**  **LBP + leg pain**  **above knee**  **LBP + leg pain**  **below knee**  **LBP + NRI** | 1.0  1.08 (0.44-2.65)  0.21 (0.05-0.95)  1 | 1.0  1.34 (0.50-3.61)  0.25 (0.05-1.19)  1 | 1.0  0.30 (0.06-1.38)  1.35 (0.49-3.71)  0.31 (0.04-2.50) | 1.0  0.28 (0.06-1.36)  1.50 (0.51-4.41)  0.33 (0.04-2.90) | 1.0  1.91 (0.70-5.21)  0.92 (0.27-3.12)  0.86 (0.17-4.28) | 1.0  3.43 (1.02-11.49)  1.35 (0.34-5.43)  1.38 (0.23-8.27) | 1.0  1.14 (0.44-2.92)  0.40 (0.11-1.46)  2.62 (0.88-7.76) | 1.0  1.0 ( 0.37-2.67)  0.33 (0.09-1.26)  2.84 (0.88-9.16) | 1.0  0.74 (0.27-2.05)  3.41 (1.46-7.97)  2.44 (0.83-7.19) | 1.0  0.52 (0.17-1.59)  2.95 (1.16-6.79)  2.07 (0.63-6.79) |

^#^ Adjusted for age, sex and educational level

*Trajectory of LBP intensity

LBP = low back pain

NRI = nerve root involvement
